# Artificial Intelligence for the Diagnosis and Management of Patellofemoral Instability: A Comprehensive Review

**DOI:** 10.3390/diagnostics15222918

**Published:** 2025-11-18

**Authors:** Michele Mercurio, Federica Denami, Andrea Vescio, Filippo Familiari, Umile Giuseppe Longo, Olimpio Galasso, Giorgio Gasparini, David H. Dejour

**Affiliations:** 1 Department of Orthopaedic and Trauma Surgery, Magna Graecia University, “Renato Dulbecco” University Hospital, 88100 Catanzaro, Italy; michele.mercurio@unicz.it (M.M.); andreavescio88@gmail.com (A.V.); filippofamiliari@unicz.it (F.F.); gasparini@unicz.it (G.G.); 2 Research Center on Musculoskeletal Health, MusculoSkeletal Health@UMG, Magna Graecia University, 88100 Catanzaro, Italy; 3 Fondazione Policlinico Universitario Campus Bio-Medico, 00128 Roma, Italy; g.longo@policlinicocampus.it; 4 Research Unit of Orthopaedic and Trauma Surgery, Department of Medicine and Surgery, Università Campus Bio-Medico di Roma, 00128 Rome, Italy; 5 Department of Medicine, Surgery and Dentistry, University of Salerno, 84081 Salerno, Italy; ogalasso@unisa.it; 6 Lyon Ortho Clinic, Clínica de la Sauvegarde, 69009 Lyon, France; dr.dejour@lyon-ortho-clinic.com

**Keywords:** artificial intelligence, deep learning, machine learning, patellofemoral instability, Rx, MRI, CT

## Abstract

Patellofemoral instability (PFI) is a multifactorial orthopedic condition affecting predominantly young and active individuals. Accurate diagnosis and personalized treatment planning remain challenging due to the complex interplay of anatomical and biomechanical factors. Recently, artificial intelligence (AI), particularly machine learning (ML) and deep learning (DL), has gained attention for its role in musculoskeletal imaging and orthopedics care. This review explores the current and potential applications of AI in diagnosis and management of PFI. A total of 11 relevant articles were identified and included in the review. Articles originated from six countries, with China having the most contributions (*n* = 4), followed by Finland (*n* = 3), and Korea, Japan, USA and Portugal with 1 each. In the results section, findings are grouped into three themes: (A) Diagnosis, (B) Outcomes and Complications and (C) Challenges, Limitations and Future Directions. The review also discussed advancements in automated image analysis, predictive modeling and outcome prediction. Overall, AI has the potential to improve consistency, efficiency, and personalization of care in patients with PFI, although still requiring technological developments for implementation in daily practice. Existing studies are limited by small datasets, methodological heterogeneity, and lack of external validation. Future research should focus on multicenter data integration, explainable AI frameworks, and clinical validation to enable translation into routine orthopedic practice.

## 1. Introduction

Patellofemoral instability (PFI) is a common orthopedic condition characterized by the abnormal movement of the patella outside the femoral trochlea, ranging from partial subluxation to full dislocation. This condition primarily affects adolescents and young active adults aged 10 to 16 years old [1,2], particularly females, and represents a significant cause of anterior knee pain, functional limitation, and recurrent joint complaints. The clinical spectrum ranges from a single episode of acute patellar dislocation to chronic instability with recurrent dislocations, often associated with a profound impact on sport participation and quality of life [3,4,5].

The pathophysiology of PFI is multifactorial, involving a complex interplay between static and dynamic stabilizers of the patellofemoral joint. Key anatomical risk factors include trochlear dysplasia, patella alta, increased tibial tuberosity-trochlear groove (TT–TG) distance, coronal plane malalignment, and rotational abnormalities of the femur or tibia [6,7,8]. It can also be associated with generalized ligamentous laxity and other co-morbidities including connective tissue disorders (i.e., Marfan’s syndrome, Ehlers-Danlos syndrome) and genetic diseases (i.e., nail syndrome, small patella syndrome) which also have multisystem manifestations [9,10,11]. Accurate diagnosis and classification of PFI are essential for guiding treatment selection and optimizing outcomes, which may include conservative rehabilitation, medial patellofemoral ligament (MPFL) reconstruction, tibial tubercle osteotomy, lateral release, and trochleoplasty [12,13]. If not treated properly, it can lead to the that is also known as interobserver variability. In recent years, artificial intelligence (AI) and deep learning (DL) applications have emerged as promising tools for enhancing objectivity, consistency, and efficiency in musculoskeletal imaging; these techniques have shown promise in automating image-based measurements, identifying pathological features, and predicting outcomes based on complex data integration [14,15,16,17]. While these technologies offer the potential to enhance diagnostic accuracy and personalize treatment strategies, their integration into clinical practice remains limited and requires further validation.

While previous reviews have highlighted AI applications across various orthopedic areas—such as anterior cruciate ligament (ACL) injuries [18,19,20], surgical planning [21], knee arthroplasty [22,23], and assessment of degenerative joint diseases [24,25]—these have largely focused on tasks like ligament tear detection, surgical guidancedevelopment of an osteoarthritic (OA) condition [26,27,28,29]. The algorithm needs to be characterized by objective, reliable, and measurable data. As stated by the Lyon school [30], the knowledge of patient’s medical and conducting a clinical examination are essential components but not sufficient; the ultimate determination relies on a thorough evaluation of both X-ray images and slices imaging. A clinical exam, including assessment of lower limb alignment, quadriceps trophism, apprehension sign and J sign, is essential for the suspected diagnosis. Radiographic examinations confirm suspected diagnosis and assess for concomitant injuries. In particular, signs such patellar height, patellar tilt and trochlear dysplasia should be evaluated with X-rays, and femoral anteversion, tibial rotation and TT-TG distance should be evaluated with computer tomography scans (CT). Otherwise, the whole description of patellofemoral abnormalities could be evaluated with magnetic resonance imaging (MRI) which is also a better option for evaluating loose bodies, assessing MPFL and localization of injury, and also plays a crucial role in the early detection and comprehensive evaluation of complications [31,32,33,34]. Overall, no single imaging parameter is sufficient for diagnosis and there may also be a clinical-radiological mismatch. In addition, current imaging techniques usually provide static information [35], which may not capture the dynamic nature of PFI. Among other limitations, knee position, flexion, and rotation during scanning can affect measurements; also, different radiologists may report different findings or measurements, a condition, implant design, and predicting postoperative outcomes. In these contexts, explainable AI frameworks have been integrated to varying degrees, with clinical validation ranging from retrospective performance assessment to limited prospective implementation. In contrast, the patellofemoral joint presents unique diagnostic and biomechanical challenges, and AI applications specifically addressing PFI have not yet been systematically reviewed.

The aim of this review is to provide a synthesis of the available evidence on AI applications specific to PFI, thus providing insights into its current role and future potential in improving patient care. To the best of our knowledge, this is the first study to comprehensively explore the applications of AI in the diagnosis and management of PFI.

## 2. Materials and Methods

**Figure 1:**
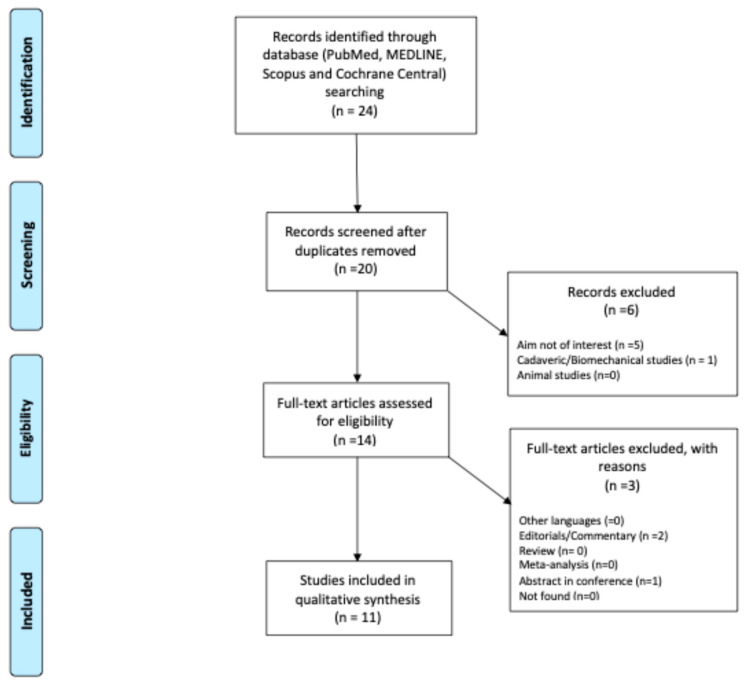
Flowchart PRISMA.

A literature review was conducted and reported in accordance with the Preferred Reporting Items for Systematic Reviews and Meta-Analyses statement (PRISMA) [36] guidelines (Figure 1). A comprehensive search of PubMed, MedLine, Scopus, and Cochrane Central was performed in June 2025. The search strategy, to retrieve relevant articles, included the following terms: “deep learning”, “artificial intelligence”, “machine learning”, “patellofemoral instability” “MRI”, “CT”, “Radiography”, “diagnosis”, and “treatment”.

Studies were included if they met the following criteria: (1) original research articles, observational studies, or clinical trials; (2) focused on AI applications for PFI; (3) published in English; (4) reported quantitative or qualitative outcomes relevant to diagnosis, management, or prediction of PFI. Exclusion criteria comprised reviews, editorials, conference abstracts without full text, and preclinical studies not involving human participants. Studies including fewer than five patients were excluded to ensure meaningful analysis. Only articles published in the last five years were considered. Two reviewers (F.D. and A.V.) independently screened records and abstracts to identify articles for inclusion, contacting a third senior author (M.M.) in cases of disagreement. The reference list of each included article, as well as the available gray literature at our institution, were screened for potential additional articles. Other reviews, editorials, letters to the editor, and expert opinions were also considered but not included. Emphasis was placed on studies applying AI to diagnosis, imaging, outcome prediction, or treatment planning for PFI. The include articles are summarized in Table 1.

A quality assessment of the methodology was carried out independently by three authors (FD, AV, and MM) using the modified Newcastle–Ottawa Quality Assessment Scale [48] (Table 2). Substantial interobserver agreement (Cohen kappa coefficients ranging between 0.59 and 0.74) was achieved. According to the overall score, the quality was categorized as “low” (0–3), “medium” (4–6), and “high” (7–9). The criteria included: 1, representativeness of the exposed cohort; 2, selection of the unexposed cohort; 3, ascertainment of exposure; 4, the proof that the outcome of interest was not observed at the start; 5, the comparability of cohorts based on structure or analysis; 6, the evaluation of the outcome; 7, a follow-up period long enough to capture the findings; 8, the adequacy of the follow-up of the cohorts. Each study received a score with a maximum of one or two points for each numbered criterion within the categories, in accordance with the modified Newcastle–Ottawa scale rules.

## 3. Results and Discussion

A total of 24 articles were identified through the initial search, resulting in 11 studies [37,38,39,40,41,42,43,44,45,46,47] that were included in the review (Figure 1). The selected articles originated from six countries, with China contributing the largest number (*n* = 4), followed by Finland (*n* = 3), and Korea, Japan, USA and Portugal with 1 each.

In the results section, findings are grouped into three thematic domains: (A) Diagnosis, (B) Outcomes and Complications, and (C) Challenges, Limitations and Future Directions.

Across the included studies, a variety of AI algorithms were applied for diagnostic and predictive purposes. DL architectures, mainly convolutional neural networks (CNNs) and hybrid DL models, were the most used and demonstrated high accuracy in automating radiographic measurements and identifying key morphological parameters associated with PFI. Traditional machine learning algorithms, including support vector machines (SVMs) were employed to predict instability risk factors and postoperative outcomes. Specifically, only two studies [37,44] reported the use of specific architecture type: in particular, VGG-16. Furthermore, three studies [38,39,41] referred broadly to the use of CNN, while two studies [40,42] specified the use of a U-Net architecture; however, these works did not provide further information on the specific model configuration or implementation details. Some authors, to mitigate constraints posed by constrained dataset sizes, used domain adaptation methodologies and approaches such as data augmentation [49]. Notably, only a subset of studies reported the full neural architecture used (e.g., VGG-16, U-Net). Several studies referred broadly to “deep learning” without specifying layer structure, training settings, or validation workflows, which limits reproducibility and comparability across models. This heterogeneity highlights the need for standardized reporting and multicenter model validation before clinical translation.

Although most models demonstrated excellent internal performance, their implementation in clinical workflows remains limited. None of the included studies reported full integration into radiology systems or clinical decision-support software; AI was primarily used as a research tool to assist image quantification or outcome prediction rather than as a real-time diagnostic aid.

**Figure 2:**
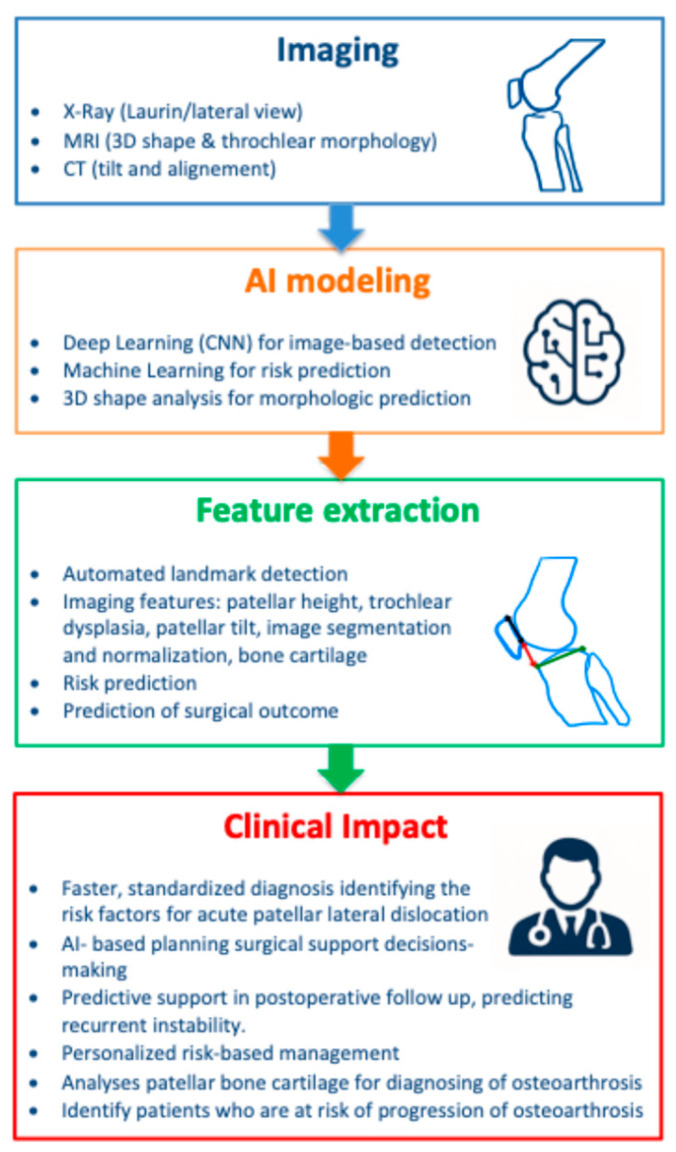
Graphical abstract summarizing AI applications in the diagnosis and prediction of patello-femoral instability (PFI). Workflow illustrating the process from imaging data acquisition through feature extraction and selection, followed by AI model application, leading to clinical decision support. The figure highlights how selected imaging features contribute to improved diagnostic consistency and treatment planning in orthopedic practice.

A schematic representation of the AI workflow for the diagnosis and management of PFI is provided in Figure 2. The diagram summarizes the data sources, feature extraction process, modeling approaches, and clinical applications discussed in this review.

### 3.1. Diagnosis

Recent advances have underscored the value of machine learning (ML) algorithms combined with optimization strategies for identifying key risk factors for PFI in pediatric and adolescent populations.

In order to mitigate operator-dependent bias inherent in the manual interpretation of radiographic images, recent studies have increasingly adopted AI algorithms to automate the quantification of critical radiological parameters, including patellar tilt and patellar height. Notably, the diagnostic performance of CNN-based model was comparable in accuracy to that of radiology specialists in the assessment of the Insall-Salvati Index (ISI), Caton-Deschamps Index (CDI), modified Caton-Deschamps Index (MCDI), and Keerati Index (KI) [26]. Ye et al. developed a CNN system for automatic patellar height, that consists of a landmark detection network (VGG16) and mathematical formulas to determine the patellar height using lateral knee radiographs [37]. Compared with the reference standard (manual measurement), the algorithm showed high accuracy in predicting the ISI, CDI, and KI (ICC = 0.91–0.95), but not the MCDI. Similarly, Tuya et al. [40] employed a deep learning method using U-Net to identify the landmarks of the patellofemoral joint (PFJ) by analyzing the Laurin. Subsequently, various parameters were determined, such as the sulcus angle (SA), congruence angle (CA), patellofemoral ratio (PFR), and lateral patellar tilt (LPT). Model performance was evaluated via the percentage of correct keypoints (PCK), intraclass correlation coefficient (ICC), mean absolute difference (MAD), root mean square (RMS) and 95% limits of agreement (LoA). Comparing the results with the mean of three radiologists, the U-Net show good performance; high ICC values (0.85–0.97), showing strong agreement with expert radiologists. The model-maintained accuracy even in cases with patellar instability or osteoarthritis. Compared to Ye et al., the U-net for segmenting landmarks using PCK at the 2 mm threshold showed larger results.

Although radiographic examinations are simple and cost-effective, their utility is limited by the lack of standardized radiographic evaluation criteria, the absence of normative reference values, and the need for proper imaging technique and views acquisition. For these reasons, other authors [41,42,43,45] have been committed to uniform the PFI assessment with MRI, demonstrating that it will be the future standard. In a 2024 study [43], researchers developed a predictive ML model using a dataset of 1.5–3.0 T MRI derived morphological measurements, including the Koshino-Sugimoto (KS) index, Wiberg classification, and TT-TG distance. One hundred and eight patients divided into two cohorts, lateral patellar dislocation (54 subjects) and control (54 subjects) groups were included in the study. The ML approach employed supervised learning techniques, training the algorithm on labeled data to distinguish between stable and unstable patellofemoral joints. Genetic algorithm-based optimization was integrated to select the most relevant features and fine-tune the model parameters, enhancing prediction accuracy and reducing overfitting. The final model demonstrated excellent diagnostic performance, with an area under the curve (AUC) of 0.934, reflecting high sensitivity and specificity in detecting patients at risk of PFI. The KS index was the main risk factor (AUC: 0.87) and, in combination with the Wiberg index (AUC: 0.85), showed the strongest association with lateral patellar dislocation. More extended trial, including the patellar height measurement, was performed by Barbosa et al. [42]. The authors suggested utilizing a deep learning algorithm (U-Net) to automatically identify the positions of landmarks and calculate index measurements in knee MRI images (both axial and sagittal). Findings indicated that the suggested approach is dependable for automatically quantifying the primary PFI indices mentioned in existing research (ISI, CDI, as well as SA, trochlear facet asymmetry (TFA), trochlear groove depth (TGD) and lateral trochlear inclination (LTI)). This method, when compared to the measurements taken by an expert musculoskeletal radiologist, significantly aids radiologists by decreasing the time required and lessening the complexity of the task involved. in terms of SA, it is the only parameter that obtained a higher intraclass correlation coefficient (ICC = 0.85–0.97 vs. ICC > 0.75) by using the U-Net-based deep learning approach develop by Tuya et al. The radiographic Laurin view offers a more detailed perspective of the trochlea, and these differences in imaging techniques might have played a role in the results that were noted. [42]. Additional femoral trochlear dysplasia (FTD) evidence was reported by Xu et al. [41], that developed a DL model to automatically detect FTD from knee MRI scans (axial image-1.5 T). Using a dataset of 464 knee MRI cases (202 with FTD and 252 normal), the authors trained a CNN with heatmap regression to identify key anatomical landmarks. The AI model’s diagnostic accuracy was 88%, with sensitivity, specificity, positive predictive value and negative predictive value ranging from 74% to 96%, outperforming junior and intermediate clinicians and matching the performance of senior experts. AI model significantly reduced diagnostic time (0.14 ± 0.11 s vs. 102.97 ± 21.26 s) compared to less experienced physicians. Furthermore, it was discovered that assessing the angle of the lateral trochlea and the depth of the trochlear groove yielded more reliable results than evaluating the ratio of the medial to lateral facets, when considering both the consistency within groups and the accuracy of disease diagnosis. In fact, MRI offers a superior view of the femoral trochlea’s structure in comparison to X-rays. On the other hand, the authors simplified the exam involving a single MRI level. As highlighted, no further information is available on multi-levels imaging or superior resolution exams. As a result, when utilizing MRI for diagnosing FTD, the choice of parameters tends to focus on assessing the angle of the lateral trochlea and the depth of the trochlear groove.

Recently, Nagawa et al. [45] integrated 3D statistical shape analysis (SSA) based on MRI with ML techniques to develop a predictive model for patellofemoral instability. According to the 3D trochlea reconstruction, the authors extracted principal components describing morphological variations that distinguish unstable knees from normal ones. Using a linear support vector machine (SVM) classifier, the model achieved an accuracy exceeding 90%, demonstrating the potential of this method to effectively discriminate anatomical features associated with the condition. The pointwise distance map revealed that the height of the trochlea in the PFI models, when compared to the normal models, was noted at the central part of the proximal trochlea floor. This approach represents a promising step toward more objective and automated diagnostic tools, with potential applications in personalized surgical planning.

Sieberer et al. developed and reported the results of a selection 2D slice methodology for calculating patellar tilt. AI was employed to analyze 3D CT scans in order to obtain patellar tilt from significant surface reference points. The findings were benchmarked against the conventional approach and the manual positioning of reference points conducted by one of the researchers involved in the study, revealing a high level of concordance between the various methods [46].

Despite promising results, heterogeneity across studies limits direct comparison of model performance.

Variations in imaging modalities (radiography, CT, MRI), preprocessing methods, and validation strategies create inconsistencies in outcome metrics. Sample sizes were generally small and single-center, often lacking external validation. Moreover, while DL models achieved superior segmentation and measurement accuracy, they remain limited by poor interpretability, whereas ML approaches, though more explainable, depend heavily on handcrafted features and smaller datasets. These discrepancies underscore the need for standardized imaging protocols, harmonized datasets, and transparent performance reporting.

### 3.2. Outcomes and Complications

While evidence regarding the application of AI in post-operative care remains limited, emerging data in the literature are promising. Recent studies have demonstrated the potential of machine learning models to predict clinical outcomes following MPFL reconstruction.

Zhan et al. developed ML algorithms that reliably forecast patient recovery and risk of complications, supporting more personalized surgical planning and postoperative management. Authors built 42 predictive models addressing seven clinical outcomes, achieving up to 97% accuracy in predicting return to pre-injury sports activity. Significantly, a low preoperative Tegner score, a reduced interval before surgery, and no severe trochlear dysplasia are key indicators for resuming preinjury sports. In contrast, the lack of severe trochlear dysplasia along with patellar alta were important factors predicting the return to pivoting sports [50]. Additionally, advanced age, being female, and a low preoperative Lysholm score strongly indicate the likelihood of experiencing recurrent instability. These predictive tools contribute to optimizing treatment strategies by integrating diverse clinical and demographic data, highlighting the growing role of AI in managing PFI [47]. A notable limitation of the available evidence is the short-term follow-up period. This is particularly relevant given the growing interest in the development of patellofemoral osteoarthritis (PFOA) as a long-term outcome following treatment for PFI, which has increasingly attracted the attention of researchers. The integration of AI-based tools, in particular DL, into outcome analysis may reasonably enhance immediate surgical results and help mitigate the risk of chronic complications, such as PFOA, through early detection and the implementation of individualized follow-up strategies.

In 2021, a study introduced a completely automated technique (CNN) for identifying PFOA in lateral view plain radiographs, based on the Multicenter Osteoarthritis Study (MOST), which is a significant multicenter dataset, employing deep learning. The developed model underwent assessment in a subject-wise stratified cross-validation framework to evaluate its reliability. The final model demonstrated a strong discriminative capacity (AUC 0.958) [38].

By analyzing large datasets that integrate imaging, kinematic, and clinical follow-up data, DL algorithms can identify early predictors of degenerative changes in the patellofemoral joint. Bayramoglu et al. [44] proposed a deep CNN (VGG-16) capable of predicting the radiographic progression of PFOA over a 7 year period using lateral knee radiographs. For purposes of comparison, a machine learning model (LightGBM—Gradient Boosting Machine, Microsoft, Redmond, Washington, USA) was developed using clinical factors such as age, gender, body mass index (BMI), the overall score from the Western Ontario and McMaster Universities Arthritis Index (WOMAC), and the tibiofemoral Kellgren and Lawrence (KL) grading. Notably, the integrated model achieved the best performance (AUC = 0.865), demonstrating its effectiveness in distinguishing patients at risk for developing PFOA and not. These findings highlight the potential of hybrid AI models in supporting early risk stratification and personalized follow-up in patients treated for PFI. In addition to predicting disease progression, Bayramoglu [39] also investigated the use of both LightGBM classifiers and deep CNN methods to predict PFOA based on texture patches analysis of lateral knee radiographs. Specifically, they compared models trained on image-based texture patches from the patellar region with those using conventional clinical data and participant characteristics. Notably, the model relying solely on texture features extracted from the superior and inferior region of interest (ROI) of the patella significantly outperformed the clinical-based model (AUC = 0.884 vs. 0.817). These results underscore the potential of patellar texture analysis as a valuable imaging biomarker for early and accurate osteoarthritis diagnosis.

### 3.3. Challenges, Limitations and Future Directions

Although not strictly categorized as AI, the work of Van Haver et al. [51] employed statistical shape modeling (SSM) to objectively characterize and classify trochlear dysplasia based on 3D CT data. By capturing shape variation through principal component analysis, the model achieved high sensitivity (85%) and specificity (95%) in distinguishing dysplastic from normal femur. This early computational approach laid important groundwork for the later development of AI-driven tools aimed at automatic morphological assessment and classification.

One of the most promising areas for future development in the evaluation of PFI lies in the integration of dynamic imaging, such dynamic measurement of PFJ alignment using weight-bearing MRI [52] or dynamic axial CT [53], and AI [54,55]. However, this is promising mostly for research because the availability of the machine is limited. Considering conventional radiological techniques limits, which provide static images of an inherently dynamic condition, there is a clear need for diagnostic approaches capable of analyzing joint behavior during movement. Emerging technologies such as cine-MRI and 4D-CT allow visualization of patellar tracking throughout flexion-extension but remain limited by technical challenges, costs, and accessibility [56]. In this context, the application of DL algorithms could enhance the analysis of these data by automating the recognition of dynamic maltracking patterns and improving the reproducibility of assessments. AI could also contribute to the development of integrated predictive models that combine morphological and functional information to support early diagnosis and personalized treatment planning. DL capable of processing sequential and temporal data (e.g., recurrent neural networks, time-series convolutional models, or transformer-based approaches) have the potential to analyze patellar tracking, alignment changes, and soft tissue interactions during movement [57,58]. By quantifying these dynamic parameters, AI could provide a more physiologically accurate assessment of instability, guide personalized rehabilitation or surgical planning, and predict postoperative outcomes. However, such applications remain in early conceptual stages and require standardized acquisition protocols, large dynamic datasets, and close collaboration between engineers and clinicians for successful translation into practice. Future research should therefore focus on the development and clinical validation of these tools to translate the theoretical potential of AI-assisted dynamic analysis into practical orthopedic applications.

Despite the growing interest and promising results regarding the use of AI in PFI, several limitations characterize the current literature [59]. First, the current body of literature remains limited, with most studies focusing on isolated applications such as automated imaging measurements or outcome prediction in small, retrospective cohorts. Large-scale, prospective studies with standardized datasets are lacking, limiting the generalizability and clinical translation of existing AI models.

Second, the heterogeneity in imaging protocols, anatomical landmarks, and diagnostic criteria across studies can hinder reproducibility and external validation of AI tools. Many models are developed using single-institution data, which may not reflect the variability encountered in broader clinical practice. On the other hand, no specific standard protocols are present regarding the number of radiographic images or patients for AI training, validation, and assessment. Standard radiographs differ in projections, patient positioning, and acquisition parameters, resulting in variable measurements of patellar height, tilt, and alignment. Similarly, MRI and CT protocols vary in slice thickness, acquisition planes, and sequences. This aspect highlights the need for a shared and standardized pathway as recently proposed with an MRI protocol for patellar height and trochlear classification [34]. Task of governmental institutions and supranational orthopedic societies should collaborate to provide guidelines for the standardization and acceptability of AI tools [35].

Another limitation should be that standard CT and MRI scans are typically acquired with the patient in a supine position, ignoring the extensor apparatus [60,61]. As a result, there is no available data on how, for example, these methods evaluate patellar tilt under weightbearing (WB) conditions, where the patella’s position may significantly differ [62]. Furthermore, definitions of anatomical landmarks and diagnostic criteria are not standardized across studies, limiting reproducibility and external validation of AI models. Recent advances in imaging techniques, such as the use of weight-bearing cone-beam CT (CBCT) with 3D spatial measurements, have demonstrated improved assessment of patellofemoral alignment under functional conditions [63]. These methods offer precise quantification of patellar tilt and shift along multiple axes, providing valuable diagnostic insights beyond traditional supine imaging. However, such analyses currently rely on manual landmark identification and geometric calculations, which can be time-consuming and operator-dependent. In this context, AI—Particularly DL—holds significant promise. Future applications may include the development of automated 3D segmentation and alignment analysis tools trained on CBCT datasets, allowing for faster and more reproducible assessment [64]. Moreover, AI-based predictive models could integrate dynamic 3D parameters with patient-specific clinical and anatomical data to enhance surgical planning and stratify the risk of recurrent dislocation.

Third, while DL models often demonstrate high accuracy, they frequently operate as “black boxes,” providing limited interpretability for clinicians. This lack of transparency can raise concerns about reliability and clinical trust, particularly in high-stakes decision-making such as surgical planning. To address this, interpretable AI approaches—such as saliency maps, attention mechanisms, and feature importance analysis—can be applied to highlight which anatomical features, measurements, or imaging landmarks drive model predictions. In our review, the studies selected focus on models that allow visualization of key patellofemoral parameters, ensuring that AI outputs provide clinically meaningful and transparent information to support surgical planning.

Fourth, there are important ethical and legal considerations surrounding the use of AI in clinical practice, including data privacy, informed consent for model use, and the potential for algorithmic bias—especially when training datasets are not representative of diverse patient populations [14,65].

Finally, the integration of AI into clinical workflows remains challenging [66]. There is a need for user-friendly interfaces, education of healthcare professionals, and robust validation through randomized clinical trials to confirm that AI-assisted tools improve diagnostic accuracy, surgical outcomes, and long-term patient care in PFI. Clinical barriers encompass limited workflow integration, lack of real-time application, and clinician skepticism due to limited interpretability of DL models. Regulatory and ethical challenges involve patient data privacy, algorithm accountability, and compliance with healthcare regulations [67,68,69]. To overcome these obstacles, future efforts should focus on developing multicenter annotated datasets, implementing explainable AI frameworks, standardizing imaging and labeling protocols, and designing AI tools that seamlessly integrate into existing clinical decision-making workflows. Addressing these factors is crucial to translate the theoretical promise of AI into routine orthopedic practice

Despite promising developments, several challenges remain in adopting AI for PFI. Future effort should prioritize multicenter collaborations to generate high-quality annotated datasets, integration of AI tools into PACS and surgical planning software [70], and clinical trials to evaluate the impact of AI on patient outcomes [71]. Interdisciplinary cooperation between clinicians, engineers, and data scientists will be essential to realize the full potential of AI in this domain.

Furthermore, ML models could be trained to distinguish between patients likely to maintain favorable outcomes and those at higher risk for complications, thereby facilitating tailored rehabilitation protocols or closer radiographic monitoring. Additional developments may also include AI-assisted dynamic simulations to model joint stress evolution post-surgery and predict long-term cartilage wear.

## 4. Conclusions

Artificial intelligence represents a promising strategy to enhance the diagnosis, classification, and management of patellofemoral instability. In this study, the comparative analyses performed between the evaluated AI models and standard radiological parameters demonstrated that the integration of automated feature extraction and predictive algorithms can improve both the consistency and reproducibility of PFI assessment. These comparisons were essential for achieving the research goals, as they quantified the added value of the proposed approach relative to conventional methods and highlighted which algorithmic components most strongly contributed to performance improvements. Current studies demonstrate potential to enhance consistency, efficiency, and personalization of care; despite these encouraging findings, clinical implementation remains limited by small, single-center datasets, heterogeneous imaging protocols, and a lack of external validation.

Future research should therefore prioritize the creation of large, multicenter datasets, the development of interpretable and transparent AI algorithms, and integration of AI tools into clinical workflows. Collaborative efforts between clinicians, data scientists, and engineers will be essential to translate these technologies from research to practice. By overcoming these barriers, AI has the potential to meaningfully support evidence-based, personalized care for patients with patellofemoral instability.

## Figures and Tables

**Table 1:** Included studies, (A) Diagnosis, (B) Outcomes and complications and (C) Future Directions and Challenges, Machine learning (ML), Artificial intelligence (AI), Convolutional neural network (CNN), Support Vector Machines (SVM), Visual geometry group 16 (VGG-16), Insall-Salvati index (ISI), Caton-Deschamps index (CDI), modified, Caton-Deschamps index (MCDI), Keerati index (KI), Patellofemoral osteoarthritis (PFOA), International knee documentation committee (IKDC), Femoral trochlear dysplasia (FTD), Kujala score (KS), Sulcus angle (SA), Trochlear facet asymmetry (TFA), Trochlear groove depth (TGD), Lateral trochlear inclination (LTI), Modified Insall-Salvati index (MISI), Caton Deschamps index (CDI), Patell trochlear index (PTI), Western Ontario and McMaster Universities osteoarthritis index (WOMAC), Congruence angle (CA), Lateral patellar tilt (LPT), Inter class correlation (ICC), Area under the curve (AUC), Receiver Operating Characteristic (ROC), and positive and negative predictive values (PPV and NPV, respectively).

N	Authors (Year) Country	Journal	AI	Sample	Aim	Result	Limits
1	Ye et al. (2020) China [37]	Eur. Radiol.	CNN (VGG-16)	1018 left knee radiographs	Determine the patellar height using lateral knee radiographs.	ISI, CDI, and KI (ICC = 0.91–0.95,) MCDI (left knee ICC = 0.65). The performance of the algorithm met or exceeded that of manual determination of ISI, CDI, and KI by radiologists.	Training size and category; lack of standard and ancillary information.
2	Bayramoglu et al. (2021) Finland [38]	Osteo. Cart.	CNN	2803 patients (19% PFOA at X-rays)	Detect PFOA from lateral view plain radiographs.	ROC AUC (0.958).	Multicenter Osteoarthritis study data alone; limited X-rays view; model explanations.
3	Bayramoglu et al. (2022) Finland [39]	Int. J. Med. Inf	CNN	5507 knees (953 PFOA)	Predict PFOA based on texture patches analysis of lateral knee radiographs.	Age, sex, BMI, WOMAC score, tibiofemoral KL grade to predict PFOA AUC (0.817).	Lack of external data.
4	Tuya et al. (2023) China [40]	Eur. Radiol.	U-Net	1431 consecutive Laurin views	Calculated radiographic parameters using the Laurin view.	SA, CA, LPT (ICC = 0.85–0.97).	Small sample size; lack of a gold standard and the inherent variation in manual measurement.
5	Xu et al. (2023) China [41]	WJCC	CNN	464 MRI 1.5 T Knee (202 FTD)	Detect FTD from knee MRI scans.	Sensitivity, Specificity, PPV and NPV of the AI model (0.74–0.96).	Single axial 1.5T MRI image.
6	Barbosa et al. (2024) Portugal [42]	Eur. Radiol.	U-Net	763 knee MRI slices (95 patients)	Index measurements in knee MRI slices (axial and sagittal).	LTI, TGD, ISI, CDI and PTI (ICC > 0.9), and SA, TFA and MISI (ICC > 0.75).	Less robust models, landmarks positioning.
7	Kwak et al. (2025) Korea [43]	KSSTA	ML	108 Patients (54 dislocated patella) (1.5–3.0 MRI)	Early diagnosis and personalized treatment planning in young patients.	KS AUC (0.87), Wiberg index AUC (0.85), IS method AUC (0.84); patellar tilt AUC (0.81) and total AUC (0.934).	Retrospective design; lack of CT and WLLRx, only logistic regression.
8	Bayramoglu et al. (2024) Finland [44]	Methods Inf. Med	CNN (VGG-16)	1832 subjects, (3276 knees)	Predict the radiographic progression of PFOA over a 7 year period using lateral knee radiographs.	AUC (0.856).	Single population trained model; No PFOA potential predictors progression consideration.
9	Nagawa et al. (2024) Japan [45]	Sci. Rep.	ML-based prediction model (SVM)	49 patients (19 PFI)	Predictive model for patellofemoral instability based on MRI.	Accuracy (0.909 ± 0.015); AUC (0.939 ± 0.009).	Small sample size; only the distal femur evaluation.
10	Sieberer et al. (2025) USA [46]	Knee	AI algorithm	60 patients (30 dislocated patella)	AI-derived measurements patellar tilt segmenting 3D CT scans.	Predicted ICC (0.86–0.90).	lack of gold standard; CT supine position.
11	Zhan et al. (2024) China [47]	Arthroscopy	ML	218 patients	Develop a ML model to predict clinical outcomes after MPFLR.	Score Accuracies Lysholm (0.884); IKDC (0.859); Kujala (0.969) Tegner (0.756).	Retrospective nature; selected knee surgery; no external validation; small sample size; short follow-up.

**Table 2:** Quality assessment of included studies according to the Modified Newcastle–Ottawa scale.

	Criteria								Total	Quality
	1	2	3	4	5	6	7	8		
Ye et al. (2020) [37]	1	1	1	1	1	1	1	1	8	High
Bayramoglu et al. (2021) [38]	1	1	1	1	1	1	1	1	8	High
Bayramoglu et al. (2022) [39]	1	1	1	1	1	1	1	1	8	High
Tuya et al. (2023) [40]	1	1	1	1	1	1	1	1	8	High
Xu et al. (2023) [41]	1	0	0	1	1	1	1	0	5	Medium
Barbosa et al. (2024) [42]	1	1	1	1	1	1	1	1	8	High
Kwak et al. (2025) [43]	1	1	1	1	1	1	1	1	8	High
Bayramoglu et al. (2024) [44]	1	1	1	1	1	1	1	1	8	High
Nagawa et al. (2024) [45]	1	1	1	1	1	1	1	1	8	High
Sieberer et al. (2025) [46]	1	1	1	1	1	1	1	1	8	High
Zhan et al. (2024) [47]	1	1	1	1	1	1	1	1	8	High

## Data Availability

The data presented in this study are available on reasonable request from the corresponding author.
